# Cancer Testis Antigen Vaccination Affords Long-Term Protection in a Murine Model of Ovarian Cancer

**DOI:** 10.1371/journal.pone.0010471

**Published:** 2010-05-12

**Authors:** Maurizio Chiriva-Internati, Yuefei Yu, Leonardo Mirandola, Marjorie R. Jenkins, Caroline Chapman, Martin Cannon, Everardo Cobos, W. Martin Kast

**Affiliations:** 1 Division of Hematology and Oncology, Texas Tech University Health Sciences Center and Southwest Cancer Treatment and Research Center, Lubbock, Texas, United States of America; 2 Departments of Internal Medicine and Obstetrics & Gynecology, and the Laura W. Bush Institute for Women's Health and Center for Women's Health and Gender-Based Medicine, Texas Tech University Health Sciences Center, Amarillo, Texas, United States of America; 3 Division of Breast Surgery, The University of Nottingham, Nottingham, United Kingdom; 4 Department of Microbiology and Immunology, University of Arkansas for Medical Sciences, Little Rock, Arkansas, United States of America; 5 Departments of Molecular Microbiology & Immunology and Obstetrics & Gynecology and Urology, Norris Comprehensive Cancer Center, University of Southern California, Los Angeles, California, United States of America; 6 Cancer Research Center of Hawaii, University of Hawaii at Manoa, Honolulu, Hawaii, United States of America; Cincinnati Children's Research Foundation, United States of America

## Abstract

Sperm protein (Sp17) is an attractive target for ovarian cancer (OC) vaccines because of its over-expression in primary as well as in metastatic lesions, at all stages of the disease. Our studies suggest that a Sp17-based vaccine can induce an enduring defense against OC development in C57BL/6 mice with ID8 cells, following prophylactic and therapeutic treatments. This is the first time that a mouse counterpart of a cancer testis antigen (Sp17) was shown to be expressed in an OC mouse model, and that vaccination against this antigen significantly controlled tumor growth. Our study shows that the CpG-adjuvated Sp17 vaccine overcomes the issue of immunologic tolerance, the major barrier to the development of effective immunotherapy for OC. Furthermore, this study provides a better understanding of OC biology by showing that Th-17 cells activation and contemporary immunosuppressive T-reg cells inhibition is required for vaccine efficacy. Taken together, these results indicate that prophylactic and therapeutic vaccinations can induce long-standing protection against OC and delay tumor growth, suggesting that this strategy may provide additional treatments of human OC and the prevention of disease onset in women with a family history of OC.

## Introduction

Ovarian cancer (OC) is the sixth most common cancer and the seventh leading cause of cancer death in women [1], [2]. Among OC, 90% of cases are represented by epithelial ovarian cancers (EOC), arising from the epithelium lining, the ovarian surface or from inclusion cysts [3], [4]. The lethality of OC stems from the inability to detect the disease at an early organ–confined stage and from the lack of effective therapies for advanced-stage disease [4]. The late diagnosis and the high rate of resistance to chemotherapy limit the treatment options available. OC patients with a family history of OC account for 10% of all cases [5]. Clinical options for these patients are surgical intervention that leads to infertility, or chemoprevention with oral contraceptives, often associated with severe side effects [6], [7]. Immunotherapy strategies including cancer vaccines are considered less toxic and more specific than current treatments [8], and therefore hold the potential to provide benefits for OC patients with evident disease and for high-risk OC patients. Because of their specificity of action, potent and lasting effects and applicability to virtually any type of tumor, anti-cancer vaccines are driving the interest of clinical oncologists. A key step in the development of basic cancer vaccines is the implementation of vaccination strategies allowing for the consistent induction of immune responses to tumor antigens. In this respect, the choice of appropriate antigens, based on both the frequency and the specificity of their expression in cancer tissues, is of paramount importance. Cancer/testis antigens (CTA) [9], [10], [11], [12], which include the Sp17 antigen [9], [13], [14], [15], [16], are emerging as promising candidates for specific immunotherapeutic targets. CTA represent a subclass of tumor-associated antigens (TAA) that are non-mutated self antigens expressed or over-expressed in tumors, and recognized by CD8 T-cells [12], [14], [17], [18], [19], [20]. The immunogenic Sp17 protein has been extensively characterized [10], [20], [21], [22], [23], [24], [25]. Human Sp17 is highly conserved, having 70% homology with rabbit and mouse, and 97% homology with baboon [25]. Sp17 has a molecular weight of 17.4 KDa, is encoded by a gene located on chromosome 11, and is aberrantly expressed in cancers of unrelated histological origin [25] including multiple myeloma (MM) and OC [21], [22]. Sp17-specific CTL, generated from normal donors, MM and OC patients, have been shown to kill HLA-matched tumor cell lines and fresh tumor cells presenting Sp17 epitopes bound to HLA class I molecules [13], [14], [21]. These observations support recent studies suggesting that Sp17 may be a suitable antigen for immunotherapy in OC [13], [25]. Recombinant proteins are commonly used in the development of antiviral vaccines, and may constitute attractive candidate antitumor vaccines [11], [26], [27], [28], [29]. Professional antigen-presenting cells (APCs) detect pathogens through a variety of receptors such as the Toll-like receptors (TLR), which recognize pathogen-associated molecular patterns, including CpG oligodeoxynucleotides (CpG ODN) within defined flanking sequences [27], [29], [30], [31]. CpG motifs, which are frequently expressed in the bacterial genome but genomically suppressed in vertebrates, are considered foreign by the immune system and, as a result, stimulate host defense mechanisms [11], [27], [29], [32], [33], [34]. CpG-ODN exhibit great potential in the therapeutic treatment of cancer due to their ability to activate innate and adaptive immunity [15], [26], [27], [28]. The TLR9-binding CpG induces secretion of Th1 cytokines, including IFN-γ and TNFα, and production of antigen-specific IgG2a by B cells [11], [12], [32], [33], [34]. In this study, we assessed the prophylactic and therapeutic immune response elicited by repeated vaccination with Sp17 recombinant protein administered with CpG. We used the murine ID8 OC cell line, derived from a spontaneous *in vitro* malignant transformation of C57BL/6 mouse ovarian surface epithelial cells to induce tumor growth in mice [34]. Our results show for the first time that priming with Sp17 protein and CpG is an effective strategy to induce durable OC therapy.

### Results Characterization of Sp17 and MHC-I expression in ID8 cells

**Figure 1:**
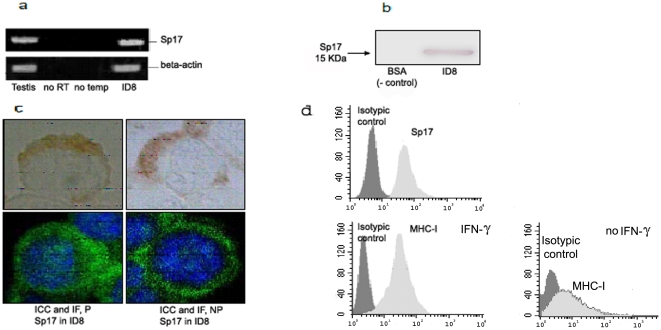
Analysis of Sp17 expression in ID8 cells. Sp17 mRNA in the murine ID8 tumor cells and in mouse testis (positive control). The mRNA levels were analyzed by RT-PCR for Sp17 expression. A cellular housekeeping gene, B-actin, was included as a control. A PCR-only control (no RT step) failed to generate a product, indicating that there was no DNA contamination in the samples. In addition to the RT-PCR (see figure **1a**), Western blot was performed and figure **1b** shows the expression of Sp17 at the protein level. The ID8 cell line was further characterized by an immunocytochemistry (ICC) and immunofluorescence (IF). This figure shows a cytospin of ID8 cells permeabilized (P) and a positive staining for Sp17 at the cytoplasm level. In addition, Sp17 was confirmed by IF in the cytoplasm. Additionally, in figure 1c, the non permeabilized (NP) cells show clear expression via IF and less by ICC. Panel d shows ID8 characterization for surface expression of Sp17 and MHC-I; isot. ctrl, isotypic control antibody; percentage indicate positive-staining cells. MHC-I expression was evaluated with or without IFN-g stimulation.

Sp17 mRNA (Figure 1a) and Sp17 protein (Figure 1b) were detected in the ID8 cell line. Further characterization by immunocytochemistry (ICC) and immunofluorescence (IF) revealed cytoplasmic and surface staining of these cells (Figure 1c). The cytospin of ID8 permeabilized (P) cells exhibits a positive cytoplasmic staining for Sp17, which was confirmed by IF. Additionally, the non permeabilized (NP) cells also show clear expression through IF (Figure 1c, lower quadrants) although less by ICC (Figure 1c, upper quadrants). We further assayed Sp17 surface expression through flow-cytometric analysis that showed high frequency of Sp17 positive cells (figure 1d). Similarly, flowcytometry analysis revealed that 60% of ID8 cells stained positive for MHC-I under basal culture conditions, while addition of 100 IU/mL IFN-γ for 72 hours resulted in 98% MHC-I positive cells.

### *In vivo* growth of ID8 cells after intraperitoneal injection

**Figure 2:**
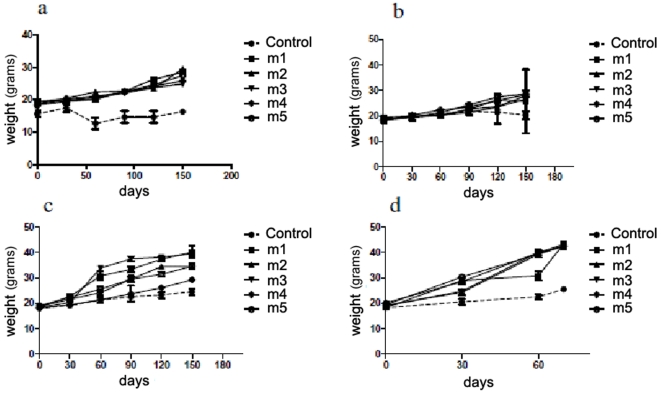
(a, b, c, d) Tumor growth in ID8-injected mice. Shows the progressive formation of ascites/tumors when i.p. injected with ID8 cells. ID8 injected mice are shown as m1-m5. Control mice were injected with PBS alone. **1a** shows mice injected with 10^5^ ID8 cells. The tumors did not reach a significant size until after 150 days. **2b** shows the progressive formation of ascites/tumors when i.p. injected with 5×10^5^ ID8 cells. The tumors did not reach a significant size until after 120 days. **2c** shows the progressive formation of ascites/tumors when i.p. injected with 10^6^ ID8 cells. The tumors reached a significant size in 90–120 days. **2d** shows the progressive formation of ascites/tumors when i.p. injected with 2×10^6^ ID8 cells. The tumors reached a significant size in 35–60 days (p<0,0001); a visible enlargement of the mice was noticed at 30 days.

**Figure 3:**
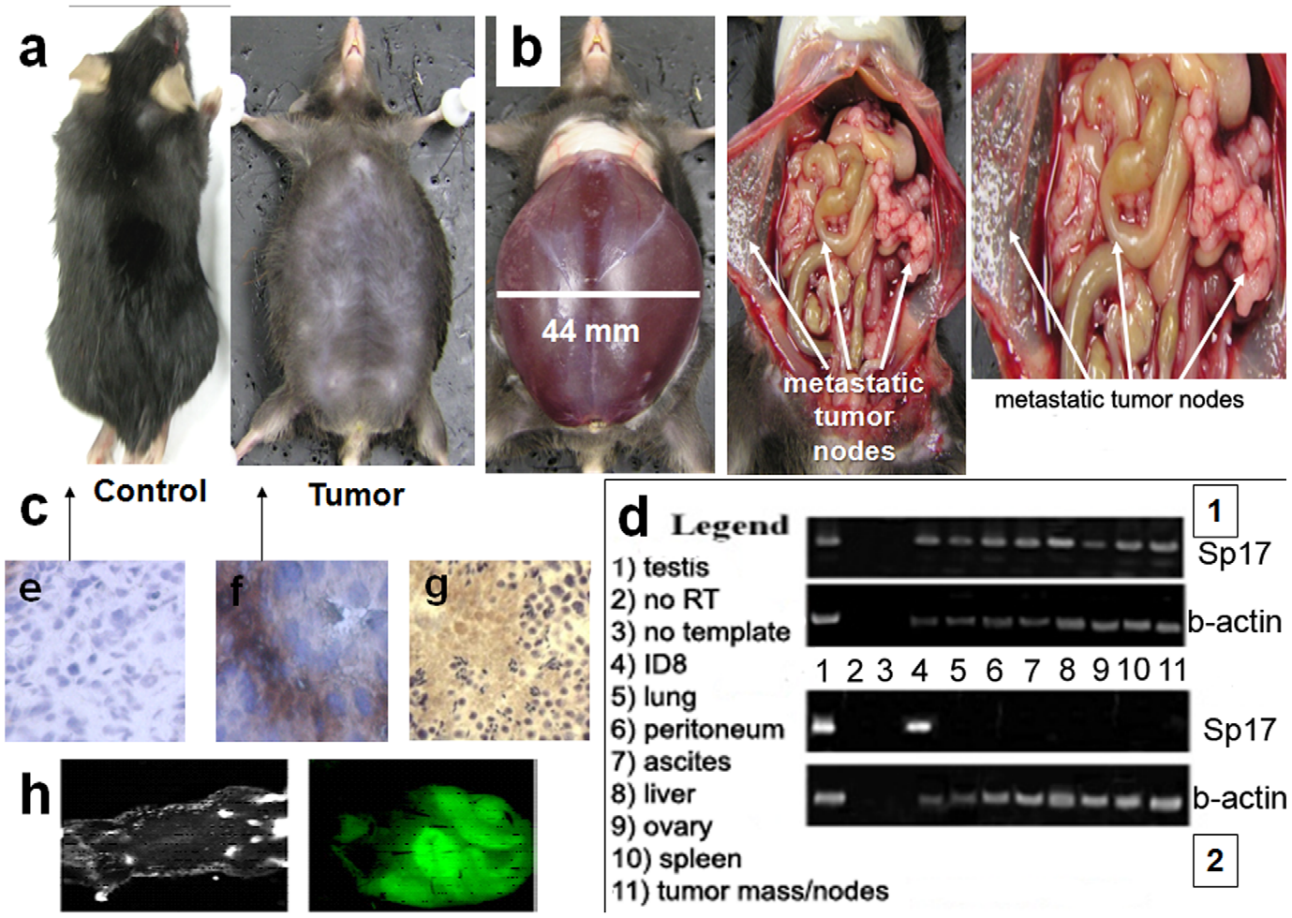
(a, b, c, d) Analysis of ID8 cells growth and dissemination in vivo. **a** shows on the left side a control mouse (width of mouse 20 mm) not injected with ID8 cells and on the right side a mouse injected with 2×106 ID8 cells after 40 days (width of mouse 44 mm). These mice represent experiments with similar results. **b** shows the full peritoneal cavity of ascites induced from an i.p injection of 2×106 ID8 in 40 days (left) and an open view of the cavity with several metastatic nodes and tumor masses (center and right, after aspirating 22 mL of ascites). **c** shows a peritoneum negative for Sp17 from a control mouse not injected with ID8 cells. **f** shows a positive expression of Sp17 on the peritoneum of a mouse injected with 2×106 ID8 cell after 40 days. Testis is the positive control for Sp17 by ICH (**g**). Figure **d** shows PCR for Sp17 DNA (and B-actin control): a tissue panel derived from (1) the organs of a 2×106 ID8 injected mouse was positive for Sp17 and a panel of tissues derived from organs of a healthy mouse revealed no expression of SP17. Positive controls (ID8 and testis) for Sp17 are also shown. Panel **h** shows in vivo fluorescence pictures of a control (left) and ID8-injected (right) mouse for the localization of GFP-positive ID8 cells.

Four different doses of ID8 cells (10^5^, 5×10^5^, 10^6^ and 2×10^6^) were injected into four groups of mice (five per group) to determine the optimal dose for induction of tumor/ascites formation. Each set of experimental inoculations with different doses of ID8 cells was performed at least three times. Animals from each tumor titration group were euthanized at different time points, at which a post mortem examination was conducted on whole animals and dissected organs (not shown). Optimal tumor growth (based on time and dimension) was observed with 1 of the 4 conditions (2×10^6^ ID8 cells) tested. Figure 2a shows that an i.p. injection of 10^5^ ID8 cells generated a tumor growth after 120 days, whilst 5×10^5^ ID8 cells generated a tumor mass/ascites in 90 days (Figure 2b). A dose of 106 cells showed a significant tumor mass/ascites growth by around 60 days (Figure 2c) and when 2×106 ID8 cells were injected, the optimal tumor mass/ascites was achieved in 40 to 45 days (Figure 2d), and most deaths occurred between 50–65 days, due either to the tumor mass or sacrifice to avoid excessive discomfort for the mice. Figure 3a depicts a mouse injected with the optimal dose (2×10^6^) of ID8 cells to generate tumor mass/ascites (44 mm in width) in less than 45 days, and a control mouse (20 mm in width) that was not injected with ID8 cells. Figure 3b shows the generation of ascites from an i.p injection of 2×10^6^ ID8 cells in less than 40 days, and also shows a detailed view of the peritoneal cavity of a mouse after aspirating 22 mL of ascites, revealing several metastatic nodes and tumor masses. Furthermore, figure 3 confirms that the cells in the peritoneum of mice injected with ID8 cells express Sp17 by immunohistochemistry (IHC), while cells from a mouse without tumor injection were negative for Sp17 (figures 3e, f and g). These results show that Sp17 is expressed in the ID8 cell line, *in vivo*. To confirm the tumor mass/ascites originated from the ID8 cells, specimens of peritoneal, tumor mass/nodes, lung, liver, ascites, spleen and ovaries were investigated by RT-PCR. Results are displayed by figure 3d, (representative picture of 5 mice per experiment, repeated in triplicate) for ID8-injected mice (figure 3d-1) and control mice (figure 3d-2). Further, a fluorescence-based localization assay was performed to detect GFP-positive ID8 cells *in vivo*, and confirmed the peritoneal localization of ID8 cells (figure 3h).

### Immunization regimens and measurement of tumor growth

The mice were vaccinated with different formulations: Sp17 only; CpG only; and Sp17+ CpG, co-administered. A total of 22 mice were immunized with each vaccine formulation. The mice were immunized intra-muscularly (i.m) with 50 µg of Sp17 protein and 20 µg CpG at different time-points as detailed above. Evaluation of tumor growth and/or ascites was monitored every three weeks using engineer calipers. Survival was followed until tumors reached volumes of more than 1,000 mm^3^, in accordance with our Institutional Animal Care and Use Committee Guidelines.

### Evaluation of survival rates after prophylactic and therapeutic Sp17/CpG vaccinations

**Figure 4:**
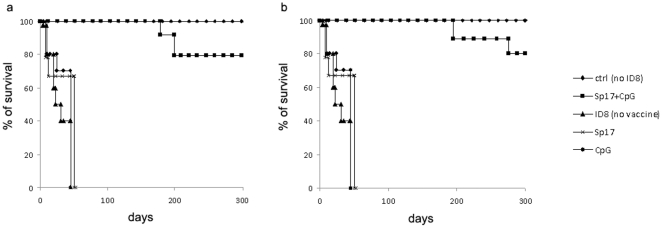
(a,b) Survival analysis with different vaccination schemes. Overall survival of mice from the four prophylaxis (**a**) or therapeutic (**b**) groups, detailed in the text (tumor free, Sp17+CpG vaccinated, unvaccinated, Sp17 or CpG vaccinated). Tumor-bearing mice were i.p. injected with 10^6^ ID8 cells for both the prophylactic and therapeutic regimens. Tumor cells were injected 30 days after the 3^rd^ vaccination of the prophylactic regimen, or 21 days before the first therapeutic vaccination. In the therapeutic group, the mean difference in weight between tumor-challenged and tumor-free animals was 10 grams at the beginning of vaccination regimen. Horizontal axis displays time expressed as days from initiation of treatment. Log-rank test indicated statistically significant difference between unvaccinated and Sp17+CpG vaccinated versus unvaccinated or CpG treated group (p<0.0001) for both regimens.

Tumor cells were injected 30 days after the 3^rd^ vaccination of the prophylactic regimen, or 21 days before the first therapeutic vaccination. All Sp17+CpG vaccinated mice (100%) were tumor-free 91 days after tumor injection with ID8 cells (2×10^6^), whereas none of the unvaccinated animals (ID8 only) were alive. Figure 4a displays the survival rates of mice that received prophylactic immunizations: 12.5% of Sp17+CpG vaccinated mice developed small tumor masses and ascites after 91 days and died 180 days after with heavy-load tumors. Moreover, 8% of the vaccinated mice developed tumors and ascites, and died after 200 days. However, those tumors were significantly smaller than the ovarian tumors of the control mice that were vaccinated with PBS only. The overall survival of Sp17+CpG vaccinated mice was 79% for over 300 days. Analysis performed by a Log-rank (Mantel-Cox) Test showed that the overall survival curves were statistically significant (p<0.0001) for the group vaccinated with Sp17+ CpG compared with the other vaccine titrations. Figure 4b shows the survival rates of mice that received therapeutic immunizations. Less than 11% of Sp17+CpG vaccinated mice developed small tumor masses and ascites after 150 days and died after 210 days of heavy-load tumors. Moreover, less than 10% of the vaccinated mice developed tumors and ascites, and died after 280 days. The overall survival of Sp17+CpG vaccinated mice was 80% for over 300 days. Analysis was performed by a Log-rank (Mantel-Cox) Test and showed that the overall survival curves were statistically significant (p<0.0001) for the group vaccinated with Sp17+CpG compared with the other vaccine titrations. Finally, control groups vaccinated with Sp17 alone and CpG alone showed some to no protection, that was not statistically significant (figure 4a and 4b).

### Measurement of Sp17-specific antibody responses and cytokine expression by ELISA assay

**Figure 5:**
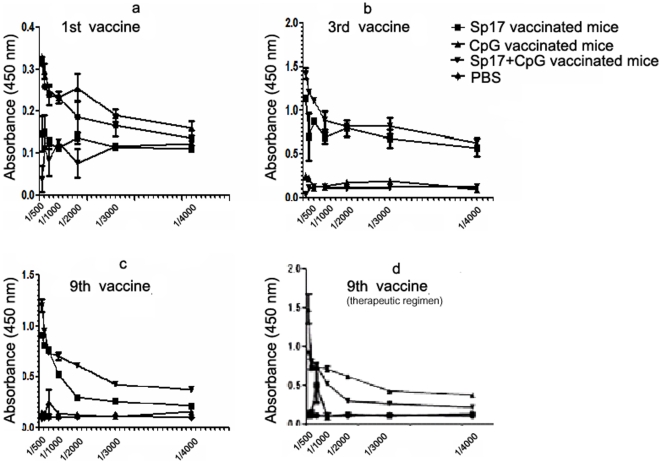
Measurement of circulating anti-Sp17 IgG following different vaccinations. Serum from mice undergone different treatments was collected and analyzed by E.L.I.S.A. for the levels of circulating anti-Sp17 IgG. The X axis shows serial dilutions of serum. a, b and c show results obtained in the prophylactic vaccination schemes, while d displays the response of mice from the therapeutic regimen. On day 7 (**a**) the immune response was low for Sp17. On day 97 (**b**), the immune response was higher than on day 7. On day 270 (**c**), the response for Sp17 was little less than on day 97.

**Figure 6:**
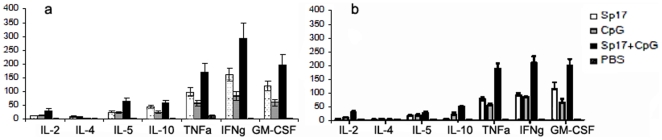
(a,b) Measurement of cytokines in mice treated with different vaccines. On day 300, serum was collected and analyzed by E.L.I.S.A. for the measurement of the indicated cytokine levels in prophylactically (**a**) or therapeutically (**b**) vaccinated mice. For both regimens, IFN-gamma, TNF-alpha and GM-CSF statistically significant increments were detected only in Sp17+CpG vaccinated mice compared with controls (PBS). No significant differences were evidenced for IL-2, IL-4, IL-5 or IL-10 levels.

Analysis of the Sp17-specific antibody response generated in the vaccinated mice is shown in figure 5. The representative ELISA assay was performed to analyze the immune response from 3 different vaccine formulations: Sp17+CpG; CpG only; and Sp17 only. Subsequent vaccinations increased specific anti-Sp17 antibody responses compared to the first vaccination. In figure 5b, we showed a high amount of specific anti-Sp17 antibodies from both Sp17 formulations at 3^rd^ vaccination. We explored the repeated vaccination schedule because, in a clinical setting, patients that are in remission are often continued to be vaccinated so that there is no recurrence of the tumor. However, there was a slight decrease of the amount of immune response compared to the 3^rd^ vaccination analyses in all three formulations at the 9^th^ vaccination (figure 5c) but anti-Sp17 IgG levels in Sp17+CpG vaccinated group were still statistically higher compared with the other vaccine formulations (p<0.001). In the therapeutic regimen group, Sp17+CpG vaccinated mice showed a significantly higher (p<0.01) production of IgG anti-Sp17 after 270 days, compared with mice treated with the other formulations (figure 5d). In figure 6 we showed the expression of cytokines at day 270 (at the 9^th^ vaccination) in both prophylactic (6a) and therapeutic (6b) regimens. Serum was collected and analyzed by ELISA assay (IL-2, IL-4, IL-5, IL-10, IFN-γ, TNF-α, GM-CSF) from mice vaccinated with Sp17+CpG and compared with serum from mice vaccinated with Sp17 alone, CpG alone or PBS alone. Interestingly, IFN-γ from the Sp17+CpG vaccinated mice was increased by almost two folds compared with the Sp17 vaccinated animals or around three folds compared with CpG vaccinated animals (figure 6a and b). In prophylactically vaccinated mice, TNF-α from Sp17+CpG formulation was increased more than three folds, as compared with the CpG vaccinated mice and only two folds compared with Sp17 vaccinated mice (figure 6a). GM-CSF increments were three folds higher than in CpG vaccinated mice and less than two folds higher versus Sp17 vaccinated mice (figure 6a). In therapeutically vaccinated animals, TNF-α from Sp17+CpG formulation was increased two folds compared with the CpG and with Sp17 alone formulations (figure 6b). GM-CSF displayed two-fold and more than one-fold increment compared with CpG and Sp17 formulations alone, respectively (figure 6b). Concerning the other cytokines, there was no significant expression of IL-2, IL-4, IL-5 or IL-10 (figure 6a and b).

### Evaluation of CTL antitumor responses

**Figure 7:**
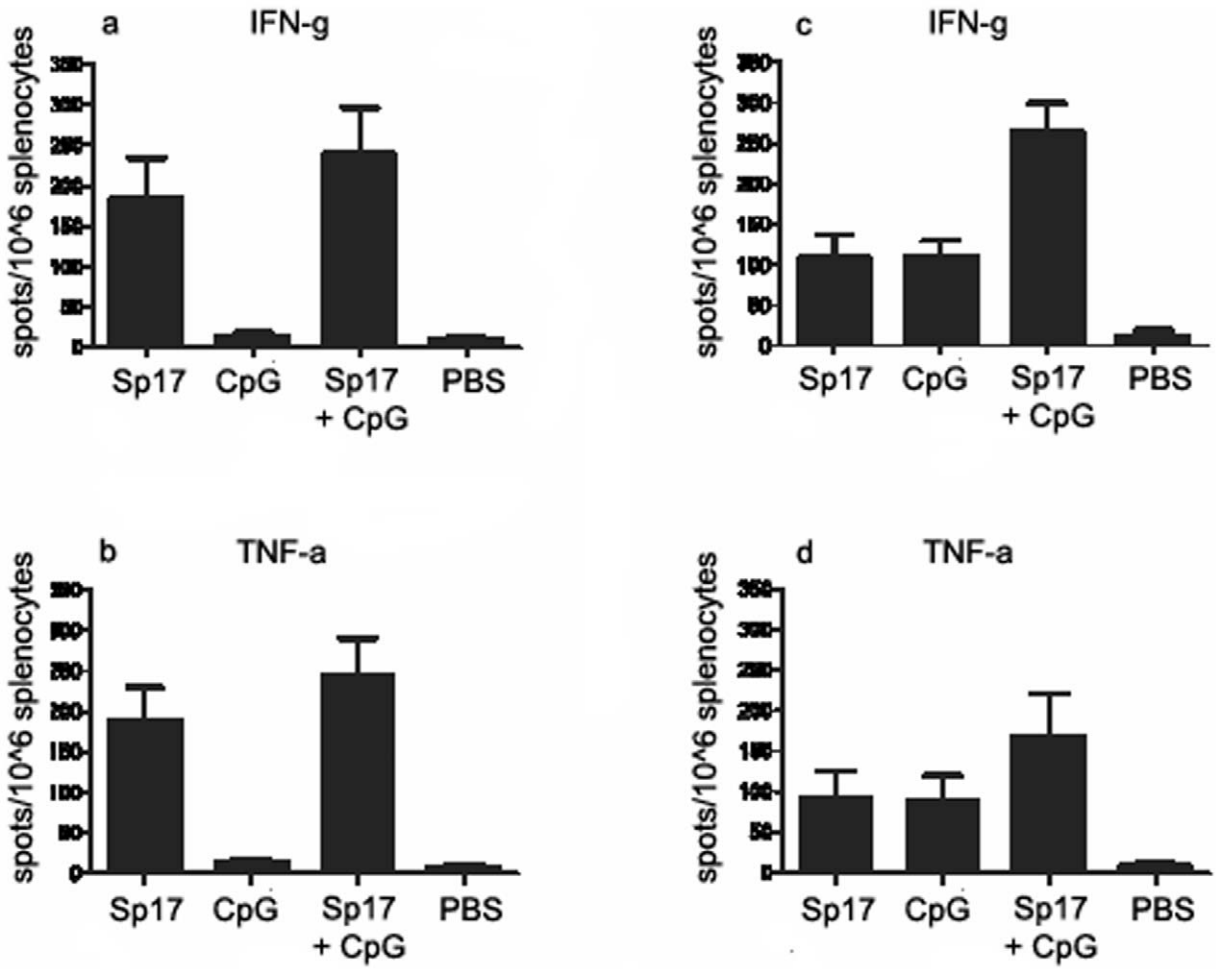
ELISPOT for the assessment of IFN-gamma and TNF-alpha serum levels. On day 270 (9^th^ vaccine) splenocytes from different formulations of vaccinated mice and controls were collected and analyzed by E.L.I.SPOT assay. **a,b**) frequency of IFN-gamma and TNF-alpha positive cells in prophylactic vaccinations; **c,d**) frequency of IFN-gamma and TNF-alpha positive cells in therapeutic vaccinations. These results are presented as spot-forming cells per 10^6^ splenocytes. Spot numbers represent the mean of ten mice in each vaccinated group; bars, SE.

**Figure 8:**
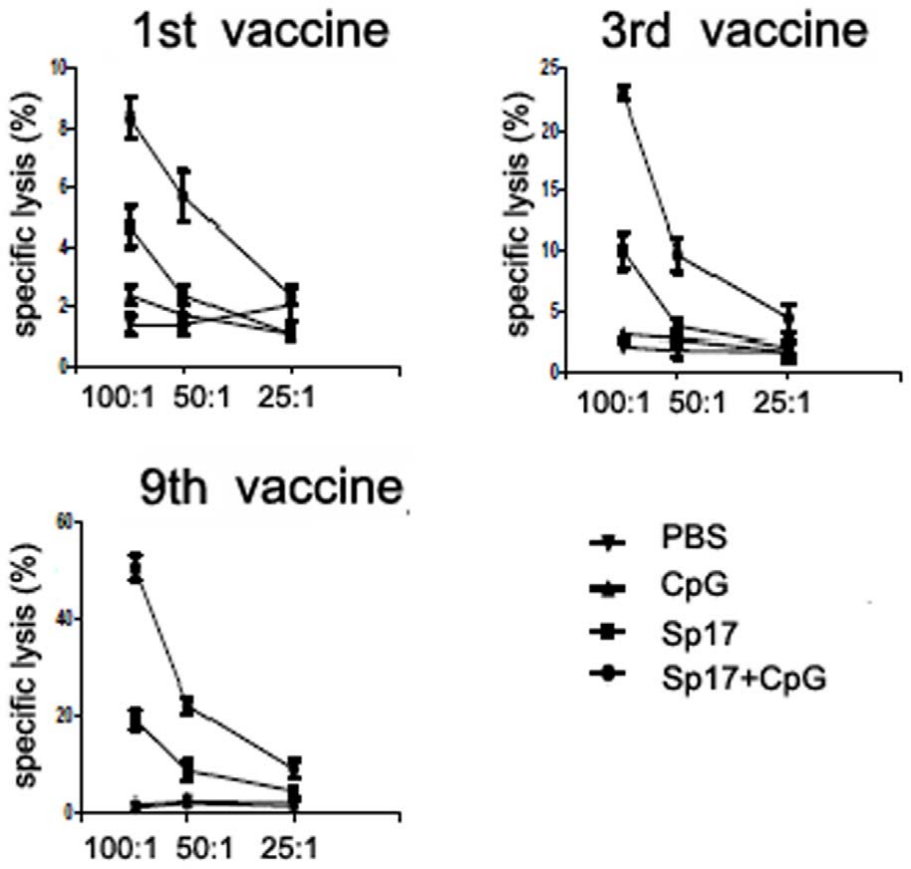
Cytotoxicity assay in the prophylaxis regimen. Splenocytes from the three different formulations of prophylactically vaccinated mice and controls were collected and analyzed by ^51^Chromium-release assay on days 7 (first vaccine), 97 (third vaccine) and 270 (ninth vaccine), using splenocytes as effector cells and ID8 as target cells. These results were obtained from three independent experiments. X axis indicate effector:target ratios.

**Figure 9:**
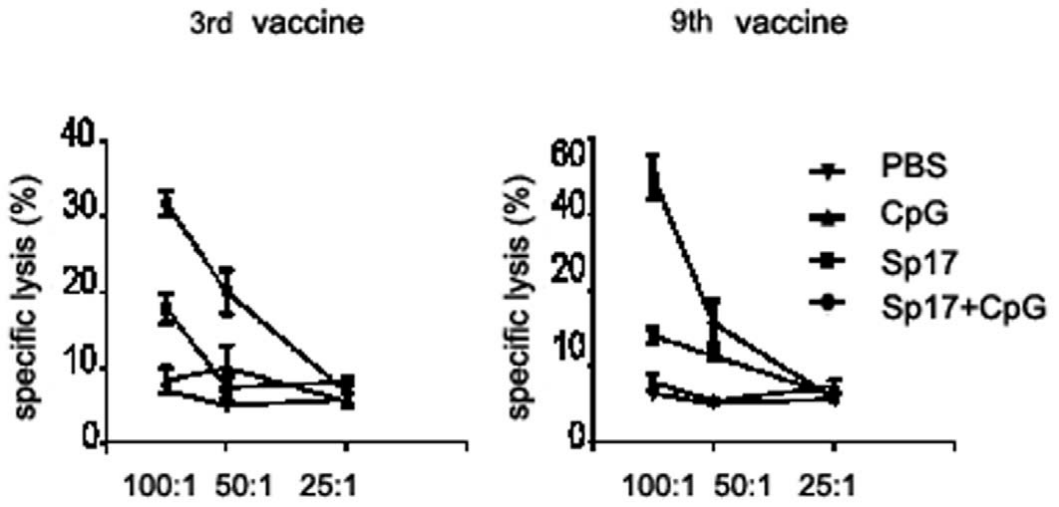
Cytotoxicity assay in the therapeutic regimen. Splenocytes from the three different formulations of therapeutically vaccinated mice and controls were collected and analyzed by ^51^Chromium-release assay. These results show three independent experiments after third vaccine (day 97) and ninth vaccine (day 270), using splenocytes as effector cells and ID8 as target cells. X axis indicate effector:target ratios.

In figure 7a, the ELISPOT IFN-γ assay was performed on day 270 (9^th^ vaccination) using spleen cells of Sp17+CpG prophylactically immunized mice. The strategy of repeated vaccinations in this animal model is to reflect current human clinical trials. These results suggest that the frequency of Sp17-specific CTL increased in the optimal group (Sp17+CpG vaccinated mice) and showed 220±30 positive spots in the spleen cells versus the Sp17 vaccinated (184±18) and versus the CpG-vaccinated mice that were as low as 9±2. In figure 7b, the ELISPOT TNF-α assay was performed on day 270 (9^th^ vaccination of prophylactic schedule) using spleen cells of Sp17+CpG immunized mice (that was the best formulation for a strong immune response). These results suggest that the frequency of Sp17-specific CTL increased in the optimal group (Sp17+CpG vaccinated mice), showing 240±35 positive spots in the spleen cells versus the Sp17 vaccinated only (200±31) and versus the CpG vaccinated mice that were as low as 12±2. Analogously, in the therapeutic vaccination regimen, the frequency of Sp17-specific CTL increased in the Sp17+CpG group with 259±20 IFN-γ and 159±30 TNF-α positive spots, versus 100±20 IFN-γ and 70±10 TNF-α positive spots in Sp17 and CpG only formulations, respectively (figure 6c and d). These results overall suggest a high and specific immune reaction induced by Sp17 when it is co-administered with CpG, regardless of the adopted vaccination schedule For the cytotoxicity assay by ^51^Cr-release measurement, splenocytes were collected at the time of the 1^st^, 3^rd^ and 9^th^ vaccinations from Sp17 vaccinated mice, CpG vaccinated mice, and Sp17+CpG vaccinated mice. The CTL assay showed stronger CTL responses in Sp17+CpG vaccinated mice compared with the mice immunized with Sp17 or CpG only, both in prophylactic (figure 8) and therapeutic (figure 9) regimens. Because in normal ID8 culture conditions *in vitro*, there is a low expression of MHC Class I molecules ID8 cells were treated *in vitro* with IFNγ (100 IU/mL for 72 hours) to induce higher expression levels, as previously reported (figure 1d, lower panel). ID8 cells activated with IFN-γ were used as better targets for the cytotoxicity assay.

### Evaluation of Th-17 and T-reg cell frequency

**Figure 10:**
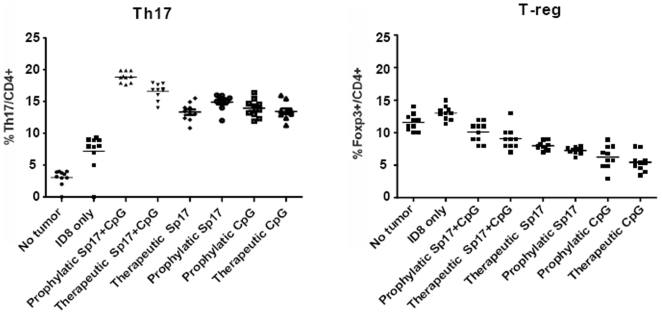
Analysis of Th-17 and T-reg population frequencies. Splenocytes from mice that received prophylactic or therapeutic vaccinations or controls (mice with no tumor or tumor-bearing mice without vaccinations) were collected at different time points (no tumor: day 0; ID8 only: day 45; prophylactic and therapeutic vaccines: day 270) analyzed by flow-cytometry for the measurement of a) Th-17 population (CD4/IL-17-double positive) or b)T-reg population (CD4/Foxp3-double positive) frequency.

Figure 10 shows the frequency of Th-17 or T-reg cells in SP17+CpG vaccinated or control mice splenocytes, collected at different time points (no tumor: day 0; ID8 only: day 45; prophylactic and therapeutic day 270).

Both prophylactic and therapeutic vaccinations elicited a significant increase in Th-17 and a decrease in T-reg cell mean frequencies (3-and 1.5-folds respectively, fig. 10) compared with tumor-challenged unvaccinated animals (unpaired t-test p<0.001, for vaccinated versus control untreated mice). Therapeutic and prophylactic vaccines consisting in Sp17 or CpG alone also induced an increment in Th17 frequencies; however, such effect was not significant (unpaired t-test p>0.05, for SP17- or CpG- only vaccinated versus control untreated mice). Sp17 or CpG administered alone reduced T-reg population frequencies at a higher extent compared with combined Sp17+CpG formulations in both therapeutic and prophylactic regimens; this reduction was significant when compared with tumor challenged unvaccinated mice (unpaired t-test p<0.01), but not when compared with Sp17+CpG vaccinations (unpaired t-test p>0.05).

## Discussion

The lethality of ovarian carcinoma primarily stems from the inability of physicians to detect the disease at the early organ–confined and usually asymptomatic stage, combined with the lack of effective therapies for advanced-stage disease [14], [33], [35], [36]. The late diagnosis and the high rate of chemo-resistance of this form of cancer proves a need for new therapeutic targets and a better understanding of the mechanisms involved in the spread of OC.

In the present study, we assessed the efficacy of Sp17 recombinant protein plus CpG in prophylactic and therapeutic settings in the murine ID8 model. Sp17 has been shown here to be expressed strongly in the cytoplasm and on surface membranes of ID8 cells (by ICC, IF and flow cytometry). We were able to follow tumor growth using Sp17 as a biomarker (figures 1 and 3). This murine model generates ascites as shown in figures 3a and 3b, and so it seems to reflect the pathology of the most aggressive and frequent form of human ovarian disease at stages III and IV. The generation of tumor nodes and fusion of the peritoneum also resemble the human process (see figure 3b), as well as the typical peritoneal localization evidenced *in vivo* by fluorescence imaging (figure 3h). The use of this animal model has been reported for different studies related to tumor targeting and vascular growth factor [23], [37], but this is the first time that a mouse counterpart of a human tumor associated antigen, Sp17, has been shown to be expressed in an OC mouse model.

One of the major criteria in deciding which candidate self-antigen to target for prevention or therapeutic strategies is creating an immune response in a safe manner, and the evidence here suggests that Sp17 may be such a candidate. We have previously shown that adoptively transferred Sp17-specific T cells have anti-tumor activity [13], [14], [21], [22], but Sp17-targeted therapeutic or prophylactic vaccination has not been tested in a tumor model. These results clearly indicate that the co-administration of Sp17+CpG i.m. injected every 30 days for a total of ten vaccinations prevented the formation of tumors up to 300 days, with a survival rate of 77% for the prophylactic group and 80% for the therapeutic vaccination group. Notably, the effects of the vaccination in the prophylactic and therapeutic regimens were similar. Both regimens induced a strong immunity that prevented tumor growth in tumor-challenged mice. The mice that ultimately succumbed to the disease showed delayed development of tumor growth compared with the unvaccinated mice. We hypothesize that our therapeutic regimen will be effective in rejecting differently staged OC, since our therapeutic vaccinations did not start until the ID8 tumors showed a significant growth, with features usually observed in human stage III-IV OC, including cancer spread beyond the pelvis, to the lining of the abdomen or to the lymph nodes (www.ovariancancer.org) [38]. Tumor-injected, unvaccinated animals displayed tumor spread to the peritoneum, lymph-nodes, lungs, liver and spleen. Indeed, the mean difference in weight between tumor-challenged and tumor-free groups was 10 grams, corresponding to about 50% mean animal weight before tumor injections (20 grams).

The activation of a strong immune response against Sp17 was demonstrated by ELISA and ELISPOT assays, showing an increase in anti-Sp17 antibodies in mice serum, expression of Th1-associated cytokines and tumor-specific cytotoxic responses. It is noteworthy that no significant serological reactivity to Sp17 was detectable before vaccination, indicating the ability of the vaccine to prime specific naïve B cells. Comparing the results of Sp-17 specific antibody titers obtained with different therapeutic vaccine formulations, we found only a small but evident increase of anti-SP17 antibody production in CpG+SP17 vaccinated animals compared with the other groups, especially following the 9^th^ vaccination. Therefore, we cannot judge whether tumor rejection following CpG+Sp17 vaccinations can be attributed to humoral responses. Given that the differences in antibody titers between groups are small, we would conclude that the tumor antigen-specific cellular immune responses we detected most probably make a larger contribution to tumor rejection in CpG+Sp17-vaccinated animals only. Interestingly, the co-administration of Sp17+CpG was better than Sp17 alone or CpG alone as an adjuvant. It has been well documented that i.m. injections of proteins generally do not induce significant immune responses unless they are mixed with adjuvants [30], [32], [39]. Effective adjuvants display at least 2 mechanisms of action: a depot effect that leads to prolonged antigen exposure in the host, and a capacity to trigger the innate immune system through activation of dendritic cells (DC) via toll-like receptors (TLRs)[35]. Upon proper antigen presentation, activated DC play a key role in the induction of T cell responses[29], [34], [40], [41], [42]. Because of their high efficacy, several recently identified TLR ligands are promising vaccine adjuvants. Synthetic ODNs containing unmethylated CpG dinucleotides flanked by two 5′ purines and two 3′ pyrimidines (CpG motif) have been reported to have immunomodulatory activities[30]. CpG motifs potently enhance T cell responses in multiple murine vaccination models[30], [31], [32], [33], [39]. By binding toll-like receptors, CpG can activate DC and macrophages to trigger the production of IL-1, IL-6, IL-12, and TNF-α, and lymphocytes, to produce IFN-γ. Overall, CpG DNA stimulates Th1-type responses, characterized by IL-12 and IFN-γ secretion with very little secretion of Th2 cytokines and a predominance of IgG2a over IgG1 in the mouse [27], [30], [32], [39], [43], [44]. We detected increased levels of TNF-alpha, INF-gamma and GM-CSF in the serum of animals treated with CpG+Sp17: this suggests a Th1 bias that is in accordance with the expected CpG adjuvant activity [27], [30], [32], [39], [43], [44]. Thus, although not formally demonstrated, the most likely source would be CD4+ Th1 T cells, and possibly CD8^+^ T cells.

Sp17 is a known cancer testis antigen [22] and has already been studied for OC T cell therapy [22], [36], showing potential application in its treatment. The cytotoxicity assays in this study showed strong anti-tumor responses in the Sp17+CpG vaccinated mice compared to the Sp17 and CpG immunized mice, both in prophylactic and therapeutic vaccinations. Vaccination with Sp17 protein in the absence of CpG resulted in weak cytotoxic responses and lack of anti-tumor effects in C57BL/6 mice indicating that CpG serves a critical role in generating effective tumor-specific cytotoxic responses and humoral responses. The killing assay showed significant differences at a high E:T ratio (100∶1); however, since the effector cells were whole splenocytes and the frequency of anti-tumor CTL in total splenocytes in the absence of a secondary *in vitro* stimulation is likely to be very low [41], [42], high E:T ratios are usually required to detect cytotoxic activity.

The Sp17+CpG vaccination did not induce significant side effects associated with inflammatory infiltration of normal tissues. Our goal was also to provide a better understanding of the role played by immunosurveillance in OC biology and progression.

Thus, we further extended our analysis to better characterize the cell-mediated anti-tumor responses elicited by Sp17+CpG vaccine. We detected a significant increase in CTL-stimulatory Th-17 cells and a decrease in immunosuppressive T-reg cells in vaccinated mice compared with non vaccinated tumor-bearing mice in both prophylactic and therapeutic regimens. This suggests that our Sp17-based vaccine formulation could have the potential to prevent the activation of immunosuppressive mechanisms that has been reported after systemic treatment with high-doses CpG and can potentially represent a major obstacle in the use of ODN-adjuvanted vaccines [40]. Although it has been recently shown that epithelial ovarian cancer-associated CD4^+^ regulatory T lymphocytes are characterized by a notable plasticity and can be reprogrammed into functional Th-17^+^ cells *in vitro* [45], it is known that Th-17^+^ and Foxp3^+^ T cells can originate from naïve CD4^+^ lymphocytes with high frequencies [46], [47], [48], [49] *in vivo*: this is in accordance with our finding that vaccines consisting in Sp17 or CpG administered alone induced a marked decrease in T-reg population frequency, with even higher degree than combined Sp17+CpG did in both therapeutic and prophylactic regimens, but were unable to significantly increase the occurrence of Th-17^+^ cells, as prophylactic or therapeutic combined Sp17+CpG. Since only Sp17+CpG injected animals efficiently rejected syngenic ID8-OC tumors, we hypothesized that a reduction in T-reg population alone without a significant increment in Th-17^+^ population is not sufficient to raise effective anti-tumor immune surveillance. Although some recent reports suggest that the presence of Th-17^+^ cells may contribute to tumor promotion [50], [51], other studies provide strong evidence that Th-17 T cell responses correlate with antitumor activity [30], [42], [52], [53], [54]. Notable studies from Restifo and colleagues have shown that adoptively transferred CD4^+^ Th17 cells were markedly more effective than CD4^+^ Th1 cells in eradication of advanced B16 melanoma in a mouse model [55]. Of particular significance, recent clinical investigation has shown that Th17 cell infiltration in ovarian tumors has a strong positive correlation with prolonged overall survival [56], an observation that stands in sharp contrast to the known association of Treg infiltration with poor prognosis and increased mortality in ovarian cancer patients [57].

Our results suggest that our vaccine formulation has the ability to redirect T lymphocyte activation from suppressor T-reg to activator Th-17 phenotype *in vivo*, in accordance with the findings by Paese and colleagues [58]. This possibility is intriguing for translation to clinical settings since in ovarian carcinoma patients tumor lesions have been shown to specifically recruit CD4^+^CD25^+^Foxp3^+^ T-reg cells, while tumor-infiltrating Th-17 cells recruit effector T cells to the tumor microenvironment and their levels positively correlate with clinical outcome. Accordingly, it has been proposed that vaccine strategies promoting Th-17 responses may achieve effective tumor control and increased survival in OC patients [54], [59]. Further, no effective prophylactic OC vaccines have been developed to date for the prevention of the disease in high-risk women. Therefore, we believe that our results provide the rationale for a paradigm shift in planning OC immunotherapy, showing that the Sp17 prophylactic and therapeutic vaccinations are capable of long-term protection against tumor onset, progression and dissemination. The innovative strategies we presented here are likely to be successfully used in tandem with standard treatment for the cure of primary and metastatic/recidivated OC and for tumor onset prevention in patients with a family history of the disease or genetic predisposition.

## Materials and Methods

### Mice

Six-week-old female C57BL/6 mice were obtained from the Jackson Laboratory (Bar Harbor, ME). Approval for the study was obtained from the local Institutional Animal Care & Use Committee. All mice were maintained in filtered-air laminar-flow cabinets under specific pathogen-free conditions. Treatment and care of the animals were in accordance with Institutional Guidelines and the Animal Welfare Assurance Act.

### Oligodeoxynucleotides

Both ODN 1826 (TCCATGACGTTCCTGACGTT) and non-CpG ODN 1982 (TCCAGGACTTCTCTCAGGTT) were phosphorothioate modified and synthesized by Invitrogen (Carlsbad, CA). ODNs were diluted in endotoxin-free water (Invitrogen).

### Construction of pQE30/Sp17 recombinant expression vectors

A primer pair, P1 (5′-GGATCC ATGTCGATTCCTTTCTC-3′) and P2 (5′-GGTACCTCAATTGTCTGCCTCTTC-3′), was designed based on the nucleotide sequence of the Sp17 mouse gene. The amplified fragment was purified and subsequently digested with Bam HI and Kpn I (Promega, Madison, WI) and ligated with vectors pQE30 to construct the recombinant vectors pQE30/Sp17 m according to standard methods. *Escherichia coli* (M15) was transformed with the resulting ligation mixture, and the transformed colonies were selected on medium containing ampicillin and kanamycin, and confirmed by sequencing.

### Expression and purification of Sp17 recombinant proteins

The recombinant Sp17 was made as previously described [25]. Briefly, *Escherichia coli*(M15) cells transformed with pQE30/sp17 m were propagated overnight in LB medium containing ampicillin (50 mg/L) and kanamycin (50 mg/L) at 37°C with shaking over night. The next day, 1 mL of the overnight culture was inoculated into 100 mL of fresh LB medium plus antibiotics and the culture was allowed to grow to an optical density of 0.6 at 600 nm absorbance. The culture was induced with 1 mM IPTG and grown for an additional 4 hours at 37°C. The cells were harvested by centrifugation at 2,000×g for 10 minutes. The recombinant Sp17 protein was purified using the Ni-NTA fast start kit (Qiagen, Valencia, CA) according to the manufacturer's protocol. The protein was tested for endotoxins and it was endotoxin-free, as assayed through the Endotoxin Colorimetric Assay Kit, HEK-Blue™ (InvivoGen). Purity was confirmed by sodium dodecyl sulfate-polyacrylamide gel electrophoresis (SDS-PAGE).

### Western blot analysis

The protein concentration from cells or tissues was quantified by Bradford assay (Bio-Rad). 25 µg of protein was resolved by SDS-PAGE, and then transferred to a PVDF membrane. After blocking, the membrane was incubated with primary mouse monoclonal anti-Sp17 antibody [22], and then washed and incubated with a horseradish peroxidase-conjugated secondary antibody (Amersham). After washing, the membrane was incubated in ECL (Enhanced Chemiluminescence, Amersham), then exposed to imaging film (Amersham) [20], [24].

### Cell lines

The murine OC cell line ID8 (kindly provided by Dr. Roby, University of Kansas) was cultured in RPMI 1640 medium supplemented with 10% fetal bovine serum and penicillin/streptomycin (10 mg/mL of each) in 5% CO_2_ at 37°C, and used within 20 passages after the initiation of the culture. Prior to injection, cells were detached from flasks by exposing them to 0.25% trypsin/PBS/EDTA for 3 minutes. ID8 cell lines were washed once and then suspended in PBS, counted, and adjusted to the appropriate densities as single cell suspensions prior to inoculation. For the evaluation of MHC-I expression, cells were cultured in the presence or in the absence of 100 IU/mL mouse IFN-γ (R&D Systems).

### *In vivo* determination of ID8 cell dose and cell injection

10^5^, 5×10^5^, 10^6^, or 2×10^6^ ID8 cells were i.p. injected into groups of C57BL/6 mice (five mice per group). Each set of experimental inoculations was performed at least three times, independently. The ID8 cells were i.p. injected at day 70 after the first tumor i.p injection. A group of control and one of tumor injected animals were euthanized 45 days later for a post mortem examination on the whole animals and dissected organs. Peritoneal tumor masses and ascitic fluid specimens were collected for further investigations.

### *In vitro* tumor cell identification

To detect ID8 cells at distal sites to that of the injection, Sp17 was identified at the mRNA level by reverse transcription-polymerase chain reaction (RT-PCR) and at the protein level by immunohistochemistry (IHC), immunofluorescence (IF) and Western blot.

### Reverse Transcription-Polymerase Chain Reaction (RT-PCR)

Total RNA was extracted from cells and organs of healthy control mice, and vaccinated and unvaccinated mice by means of Tri-reagent (Sigma, St Louis, MO). All total RNA specimens were treated with 5 µg of RNase–free DNase I (Promega) at 37°C for 2 hours. mRNA was then separated by using Oligotex mRNA Mini Kit (QIAGEN, Valencia, CA). First-strand complementary DNA (cDNA) synthesis was performed by using oligo (dT) 15 primers that amplify cDNA of approximately 500 base pairs (bp). The PCR primers for Sp17 were as follows: 5′-GGCAGT TCT TAC CAAGAAGAT-3′ and 5′-GGA GGT AAA ACC AGT GTC CTC-3′. PCR was performed by means of 35 amplification cycles at an annealing temperature of 57°C. Two positive control amplifications (containing the cDNA of testis and the pQE30/Sp17 plasmid) and negative controls for the PCR reaction mixture (water) were also performed each time. RNA integrity in each sample was checked by PCR amplification of a β-actin gene segment. Successful removal of genomic DNA contamination was confirmed in each sample by amplification of the RNA without prior reverse-transcription. PCR products were visualized on an ethidium bromide agarose gel for a DNA band of the expected size, using an ultraviolet light trans-illuminator. All results were confirmed by four independent RT-PCRs.

### Immunization and tumor challenge

Female C57BL/6 mice (6 weeks old) were immunized i.m. with 50 µg Sp17 protein (in 20 µl of sterile water) and 20 µg CpG (in 20 µL of sterile water). The mice were injected every 30 days for a total of 300 days. As control groups, the mice were vaccinated with 50 µg Sp17 protein only or 20 µg CpG only. 70 days after the first vaccination, mice were challenged i.p. with the optimal dose of 2×10^6^ ID8 murine OC cells. Survival was followed until tumors reached volumes of >1,000 mm^3^.

### Immunohistochemistry (IHC)

Tumor tissue was treated by the freezing tissue procedure, or tissue in paraffin, and 30% of the tissue organs were prepared as single cell suspensions and stored at −20°C [30]. After deparaffining and re-hydration, antigen retrieval was performed in a thermostatic bath (Fisher Scientific, Pittsburgh, PA) at 98°C for 30 minutes in a freshly prepared 1 mM EDTA solution. After 15 minutes incubation in a 3% H2O2 solution, the sections were exposed for 1 hour at room temperature to the primary mouse monoclonal anti-human Sp17 antibody [17], [22], diluted in TBS + BSA (0.2%) + NaN_3_ (0.02%), or to 1 mg/mL mouse IgG1 (DAKO, Carpinteria, CA) as a negative control. After washing 3 times for 5 minutes in TBS + Tween 20%, sections were incubated for 30 minutes with the secondary antibody (Envision system, DAKO) followed by 5 minutes dark incubation with DAB system (DAKO), performed to visualize brown precipitates as reaction results. Cells were counter-stained with hematoxylin (Fisher Scientific) and results were evaluated by light microscope [30].

### Immunocytochemistry/Immunofluorescence (ICC/IF)

A standardized technique for detecting Sp17 in ID8 cells was performed as previously described [21], [22]. Briefly, an ID8 single cell suspension was counted (5×10^4^ cells/100 µL) and washed with PBS. Afterwards, slides were set up with a filter card and introduced into the Shandon Cytospin-2 and spun at 800 rpm for three minutes. The funnel and filter were removed from the glass slides, fixed with SlideRite (Fisher Scientific) and the cells were allowed to air-dry overnight. 5×10cells were permeabilized with 0.5% Triton X-100 (Sigma Ltd, St. Louis, MO, USA) 0.1% sodium citrate in PBS at 4°C for 15 minutes. Cells were then treated with either primary mouse antibodies raised against human Sp17 (mouse monoclonal anti-human Sp17 antibody [30], dilution 1∶400 in PBS) at room temperature for two hours, or with 1 mg/mL mouse IgG1 (DAKO) as a negative control. This was followed by 30 minutes incubation with the Envision System (DAKO). The DAB system (DAKO) was used to yield brown reaction products in the case of ICC, while FITC conjugated rabbit IgG secondary antibody (Abcam, Cambridge, MA) was used to bind the primary antibody for IF. The immunocytochemical reactions were observed using a light microscope [30]. For IF, results were analyzed using an Olympus IX71 inverted microscope equipped with a Fluoview 300 confocal laser system (Olympus, Center Valley, PA).

### Cytotoxicity assays

Standard 4-hour ^51^Cr-release assays were performed to determine the cytotoxic activity of the Sp17-stimulated splenocytes. The ID8 target cells endogenously express Sp17. All experiments were set up in quadruplicates and repeated at least three times. Standard deviations (SD) were determined based on the quadruplicates. For all targets, cell viability was >90% with the maximum release in excess of 2000 cpm and the spontaneous release <30% of the maximum release.

### ELISPOT- CD8 T cell assay

Immune responses generated by the vaccines were measured using ELISPOT assays to detect CD8 T cells secreting IFN-γ (Mabtech, Inc., Mariemont, OH) using purified CD8 T cells (Miltenyi Biotech, Auburn, CA). Serial dilutions of CD8 T cells were tested against 3×10^6^ stimulator cells. Spot counting was done with an AID ELISPOT Reader System (Cell Technology, Inc., Columbia, MD).

### ELISPOT IFN-γ and TNF-α Assays

Cytokine expression by T cells from the immunized animals was evaluated using the ELISPOT assay (U-CyTech, Utrecht, The Netherlands) according to the instruction manual. Briefly, the 96-well filtration plates (Millipore, Bedford, MA) were coated with 100 µl diluted antibodies. After overnight incubation at 4°C, the wells were washed and blocked with washing and blocking buffer. T cells from the spleens of vaccinated mice (3×10^6^ cells/mL) were added to triplicate wells and incubated with 20 µg/mL Sp17 protein at 37°C in an atmosphere of 5% CO_2_ for 48 hours. Positive control wells were added with Con-A (5 µg/mL), and background wells were added with RPMI 1640 medium. The plates were then washed extensively (10 times) and incubated with 100 µl biotinylated detection antibodies at 4°C overnight. After washing six times, 50 µl diluted GABA was added and incubated for 1 hour at 37°C and then washed twice. The spots were developed by adding 30 µl Activator I/II solution and incubating at room temperature for 25–30 minutes. Spot counting was done with an AID ELISPOT Reader System (Cell Technology, Inc., Columbia, MD).

### ELISA for Sp17 antibodies

Animals were vaccinated every 30 days and their blood samples were collected before each injection. Briefly the 96-well plates were coated with Sp17 recombinant protein (5 µg/µl) and incubated overnight at 4°C. After washing and blocking, the goat Sp17 polyclonal antibody as positive control or serial dilution of mice sera were added and incubated at 37°C for 1 hour. After washing with PBS/0.05% Tween-20, HRP-conjugated rabbit anti-goat antibody (Abcam) was added and allowed to incubate at 37°C for 1 hour. The reaction was developed by adding TMB Microwell substrate and stopped by 2 M H2SO4. The absorbance was read at 450 nm.

### ELISA for cytokines of the sera

Cytokine concentration determined from standard curves. Sera from vaccinated mice were collected to measure Sp17 levels. Serum cytokine levels were measured by using commercial ELISA kits (R&D Systems, Minneapolis, MN), in accordance with the manufacturer's instructions. Briefly add 50 uL of Standard, Control, or sample per well. Incubate for 2 hours at room temperature. Plate layouts are provided to record standards and samples assayed. After the last wash, add 100 uL of mouse cytokines Conjugate to each well and Incubate for 2 hours at room temperature. Repeat the aspiration/wash, then add 100 uL of Substrate Solution to each well. Incubate for 30 minutes at room temperature. Add 100 uL of Stop Solution to each well. Gently tap the plate to ensure thorough mixing. Determine the optical density of each well within 30 minutes, using a microplate reader set to 450 nm.

### Flow-cytometry

Flow-cytometric analyses were performed through BD FACSCanto™ II Flow Cytometry System (BD Biosciences) and CellQuest software. For analysis of ID8 cells, exponentially-growing cells were detached with trypsin for 5 minutes, washed twice in PBS supplemented with 1% BSA and fixed with 2% paraformaldehyde at room temperature for 10 minutes. Then, cells were washed twice in PBS/BSA and allowed to incubate for 1 hour on ice with anti-MHC-I antibody (Abcam) and mouse anti-SP17 monoclonal antibody (developed in our lab). For analysis of splenocytes, cells were washed twice in PBS, fixed with 2% paraformaldehyde at room temperature, and then permeabilized with 0.5% saponin for 10 minutes on ice. After washing with PBS, anti-CD4-PE, Foxp3-PE (Abcam) and IL-17-Alexa Fluor® 700 (BD Biosciences) were added and allowed to incubate for 1 hour on ice. Then, cells were washed twice with PBS and analyzed.

### Statistical analysis

Tumor growth and chromium release assay, ELISPOT assay, ELISA assay and flowcytometry data were analyzed by a one-tailed, paired Student's test and survival rates were analyzed by log-rank test. All statistical analyses were performed through GraphPad Prism 5® (GraphPad Software, Inc., La Jolla, CA).
